# Hyaluronic Acid-Based Nanocapsules as Efficient Delivery Systems of Garlic Oil Active Components with Anticancer Activity

**DOI:** 10.3390/nano11051354

**Published:** 2021-05-20

**Authors:** Małgorzata Janik-Hazuka, Kamil Kamiński, Marta Kaczor-Kamińska, Joanna Szafraniec-Szczęsny, Aleksandra Kmak, Hassan Kassassir, Cezary Watała, Maria Wróbel, Szczepan Zapotoczny

**Affiliations:** 1 Jagiellonian University, Faculty of Chemistry, Gronostajowa 2, 30-387 Krakow, Poland; mal.janik@uj.edu.pl (M.J.-H.); kaminski@chemia.uj.edu.pl (K.K.); ola.kmak@student.uj.edu.pl (A.K.); 2 Jagiellonian University Medical College, Faculty of Medicine, Chair of Medical Biochemistry, Kopernika 7, 31-034 Krakow, Poland; marta.b.kaczor@uj.edu.pl (M.K.-K.); mtk.wrobel@uj.edu.pl (M.W.); 3 Jagiellonian University Medical College, Faculty of Pharmacy, Department of Pharmaceutical Technology and Biopharmaceutics, Medyczna 9, 30-688 Krakow, Poland; joanna.szafraniec@uj.edu.pl; 4 Medical University of Lodz, Department of Haemostasis and Haemostatic Disorders, Chair of Biomedical Sciences, Mazowiecka 6/8, 92-215 Lodz, Poland; hkassassir@cbm.pan.pl (H.K.); cezary.watala@umed.lodz.pl (C.W.); 5 Institute of Medical Biology, Polish Academy of Science, Lodowa 106, 93-232 Lodz, Poland

**Keywords:** encapsulation, drug delivery, hyaluronic acid, oil-core nanocapsules, garlic oil, diallyl disulfide, diallyl trisulfide, glutathione, sulfurtransferases, sulfane sulfur

## Abstract

Diallyl disulfide (DADS) and diallyl trisulfide (DATS) are garlic oil compounds exhibiting beneficial healthy properties including anticancer action. However, these compounds are sparingly water-soluble with a limited stability that may imply damage to blood vessels or cells after administration. Thus, their encapsulation in the oil-core nanocapsules based on a derivative of hyaluronic acid was investigated here as a way of protecting against oxidation and undesired interactions with blood and digestive track components. The nuclear magnetic resonance (^1^H NMR) technique was used to follow the oxidation processes. It was proved that the shell of the capsule acts as a barrier limiting the sulfur oxidation, enhancing the stability of C=C bonds in DADS and DATS. Moreover, it was shown that the encapsulation inhibited the lysis of the red blood cell membrane (mainly for DADS) and interactions with serum or digestive track components. Importantly, the biological functions and anticancer activity of DADS and DATS were preserved after encapsulation. Additionally, the nanocapsule formulations affected the migration of neoplastic cells—a desirable preliminary observation concerning the inhibition of migration. The proposed route of administration of these garlic extract components would enable reaching their higher concentrations in blood, longer circulation in a bloodstream, and thus, imply a better therapeutic effect.

## 1. Introduction

Nowadays, there is increasing interest in and need for the development of chemoprevention against cancer that includes also the application of phytochemicals, which are dietary natural compounds. Their anticancer action may be realized through the prevention, delay or inhibition of cancerogenesis. The biological and medical impact of plant compounds has been proven by molecular and epidemiologic studies showing a significant influence of diet on anticancer effects [1,2,3,4]. There are numerous anticancer phytochemicals, including fatty acids, vitamins, antioxidant phenolic, dietary fiber, flavonoids, polyphenols etc. Herein, we focus on garlic compounds, the phytochemicals with a number of beneficial pro-health effects (i.e., anticancer, antifungal, antimicrobial, antithrombotic, supporting cardiovascular disease treatment, stimulating the immune system, etc.) [5,6]. Garlic active compounds (organosulfur compounds such as diallyl sulfide (DAS), diallyl disulfide (DADS), diallyl trisulfide (DATS), diallyl thiosulfonate (allicin), E/Z-ajoene, S-allyl-cysteine (SAC) or S-allyl-cysteine sulfoxide (alliin), among others) inhibit cancer cell growth [7], and induce caspase-dependent [8] and independent [9] apoptosis in different cancer cell lines. Additionally, some chemicals mentioned above (e.g., DADS, DATS, SAC) are well-known as sulfane sulfur precursors [10,11,12]. The sulfane sulfur atom occurs in compounds at the 0 or −1 oxidation state and is always covalently bound to another sulfur atom. This type of atom is highly metabolically active and can be easily removed from the structure of compounds. Sulfane sulfur-containing compounds are formed in mammalian cells during the non-oxidative l-cysteine metabolism pathway. They are endogenous metabolites formed from cysteine and/or homocysteine in the processes of being catalyzed by cystathionine γ-lyase (CTH, EC 4.4.1.1). Sulfurtransferase 3-mercaptopyruvate (MPST, EC 2.8.1.2) and rhodanese (TST, EC 2.8.1.1) are sulfane sulfur carrier proteins. Studies on the biological role of these bioactive compounds are hindered by exceptional reactivity and instability of these structures. Hence, the search for exogenous, naturally occurring precursors of sulfane sulfur is of fundamental importance. However, the greatest challenge is to find a suitable carrier that will increase the bioavailability of these compounds without damaging their structure.

The challenge of delivering oily, lipophilic therapeutics including DADS and DATS is one of the major pharmaceutical problems. Most studies focused on the molecular effects of these compounds report dissolving DADS [13,14] and DATS [15] in dimethyl sulfoxide (DMSO). However, during the last few decades, there have been an increasing number of works considering the delivery of hydrophobic actives using drug-delivery systems (DDSs) based on, e.g., polymers, including e.g., polysaccharides [16,17,18,19,20], liposomes [21], bilosomes [22]. In particular, the nanoscale systems are of high interest for the encapsulation of hydrophobic pharmaceuticals and nutraceuticals [23,24,25]. The literature reports some systems with DADS and DATS, i.e., solid lipid nanoparticles [26], mesoporous silica nanoparticles [27], oil-free microemulsion [28] or water-based emulsifying system based on corn oil and surfactant [29].

Furthermore, except from the problem of solubility, various natural compounds, including garlic oils, often exhibit instability in normal or close to normal conditions, e.g., in oxidizing or reducing conditions (e.g., glutathione) [10], in the presence of H_2_O_2_ [30], antioxidants or different buffers [31], in blood or under simulated digestive conditions [32] or at higher temperatures [31,32,33,34]. Thus, the hemolytic effect on red blood cells, possible interaction with serum components (e.g., proteins) or with a digestive system environment (e.g., specific conditions like different values of pH, presence of various enzymes) need to be considered as possible side-effects during in vivo application of such compounds. This is of particular importance, because the hemolytic potential or interaction (aggregation) with other blood components was found to correlate with the intensity of lesions [35,36].

To address these challenges, the study presented herein includes the encapsulation of sparingly water soluble natural garlic oil compounds (DADS and DATS) aiming mainly at effectively carrying them in physiological environments and protection against oxidation for safe storage and therapeutic action. The formulation is based on nanocapsules with a hyaluronate shell surrounding liquid oil cores dispersed in an aqueous medium [19,37,38]. The hydrophobically modified natural polysaccharide, the sodium salt of hyaluronic acid (HA), constitutes a shell that stabilizes the nanodroplets by the polymeric hydrophobic chains anchoring in the oil core. The degradation of unstable DADS and DATS prevented through the polysaccharide shell was characterized by the study of the oxygenation impact. Furthermore, since the metabolic transformation pathways of DADS and DATS are known [10], it was tested whether the encapsulation process of these compounds causes a loss of their biological function. For this purpose, we used non-oxidative metabolism of l-cysteine, as well as the study of the level of low molecular weight sulfur-containing compounds (glutathione and cysteine). The anticancer properties of studied compounds were tested in vitro including cytotoxicity and the influence on cancer cells’ migration. Since the formulated nanocapsules with enclosed drugs potentially may be delivered into the bloodstream, the basic observations including interaction with human serum and red blood cells are described. Non-encapsulated DADS and DATS exhibit toxic effects on red blood cells triggering oxidative hemolysis [39]. Importantly, the long-term stability of nanocapsules with encapsulated DADS and DATS was proven by monitoring their zeta potentials and sizes within a period of a few weeks, which confirmed our previous observations regarding the durability of tested nanocapsules over time [19,37,38].

## 2. Materials and Methods

### 2.1. Preparation of Nanocapsules

**Figure 1:**
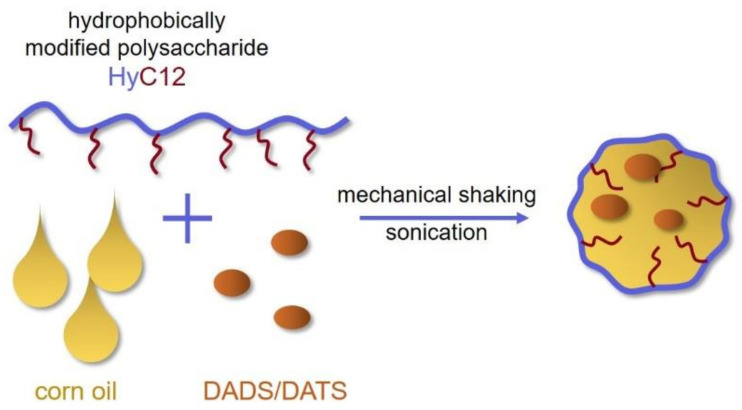
Scheme illustrating preparation of the nanocapsules with diallyl disulfide (DADS) or diallyl trisulfide (DATS) cargo molecules.

Nanocapsules based on the dodecyl derivative of hyaluronic acid sodium salt (HyC12) were prepared in ultrasound-assisted emulsification (Figure 1) according to the previously described procedures [37]. It is a surfactant-free method based on an ultrasound-assisted emulsification process. Briefly, the aqueous solution of the dodecyl derivative of HA (HyC12, DS = 2%, see synthesis details in [37]) (1 g/L in phosphate-buffered saline (PBS, Sigma-Aldrich, Poznan, Poland, tablets: 0.01 M phosphate buffer, 0.0027 M KCl and 0.137 M NaCl, pH 7.4, at 25 °C)) was mixed with an appropriate oil phase in 1000:3 (*v*/*v*) ratio. The used oils phases enabled to obtain three different types of nanocapsules: corn oil core (capsCO) (CO, Sigma-Aldrich, Poznan, Poland), DADS (≥98% HPLC, Sigma-Aldrich, Poznan, Poland) in CO (560 g/L, capsDADS) and DATS (≥98% HPLC, Sigma-Aldrich, Poznan, Poland) in CO (560 g/L, capsDATS). The mixtures were homogenized using a vortex shaker (IKA, Königswinter, Germany) for approximately 20 min, followed by the 30 min sonication in an ultrasonic bath (540 W, Sonic-6, Polsonic, Warsaw, Poland).

### 2.2. Stability of Diallyl Disulfide (DADS) and Diallyl Trisulfide (DATS) in Oxidizing Conditions

The stability of DADS and DATS in oxidizing conditions was studied by nuclear magnetic resonance (^1^H NMR)-based experiments. Nanocapsules were prepared as described with one change: HyC12 was dissolved in deuterium oxide (D_2_O, 99 atom % D, Sigma-Aldrich, Poznan, Poland). Nanocapsules and non-encapsulated oils were treated by O_2_ generated by the oxygen concentrator (DeVilbiss Healthcare, Port Washington, NY, USA). For DADS and capsDADS it was 3 times for 30 min while for DATS and capsDATS it was 3 times for 60 min. After oxygenation of oils, samples were dissolved in chloroform-d (CHCl_3_-d, ≥99.8% D, 0.03% (*v*/*v*) tetramethylsilane (TMS), Sigma-Aldrich, Poznan, Poland) (DADS/TS-O_2_ in CHCl_3_) and D_2_O (DADS/TS-O_2_ in D_2_O) and intensively shaken. Non oxygenated oils were dissolved in CHCl_3_-d and D_2_O as well. Samples were characterized using a Bruker Avance III HD 400 MHz (Bruker, Billerica, MA, USA). Signal at ~4.8–4.95 ppm belonging to D_2_O, and those at ~7.5 ppm, ~7.2–7.3 ppm, ~7.0 ppm, ~2.15 ppm, ~1.5 ppm, and ~0.0–0.1 ppm belonging to CHCl_3_-d and its impurities were cut out. The singlet at ~1.3 ppm, which also belongs to CHCl_3_-d (or its impurities) was not removed as it was overlapped by the other signals.

### 2.3. Hemolysis Assay

The toxic effect of encapsulated DADS, DATS and CO on erythrocytes was investigated by the in vitro hemolysis assay based on measuring the release of hemoglobin from destroyed red blood cells (RBCs) [36].

Human blood samples were received from 8 healthy donors (aged 20–60 years) and collected into a vacuum tube containing 0.105 M buffered sodium citrate (BD Vacutainer^®^ blood collection tubes, BD, Franklin Lakes, NJ, USA), with the final citrate:blood ratio of 1:9 *v*/*v*. Samples were washed three times with cold PBS (pH = 7.4) and centrifuged at every stage (5 min, 1000× *g*, Sigma 2-16K centrifuge, Polygen, Wrocław, Poland). After the last washing, centrifuged RBCs were diluted in PBS to 2% suspension (*v*/*v*). The samples: PBS (as a negative control), H_2_O (as a positive control), CO, DADS and DATS in PBS, encapsulated corn oil (capsCO), encapsulated DADS (capsDADS) and encapsulated DATS (capsDATS), were supplemented at the volumetric ratio of 3:2 with aliquots of the stock RBC suspension to give a final concentration of 2% (*v*/*v*) (each sample prepared in triplicates). Samples were mixed and incubated at 37 °C under gentle shaking for 1 h. Following this, samples were centrifuged at 5000 rpm for 5 min and the supernatant from the upper 2/3 volume above the sedimented RBC was harvested. The absorbance of the hemoglobin released into the supernatant was measured at 540 nm using a Perkin Elmer Victor x4 2030 multilabel reader (Waltham, MA, USA). The hemolysis ratio was calculated for the averaged triplicate readings as a percentage of the complete hemolysis, using the following equation:

(1) $$hemolysis\%=\frac{Abs_{sample}-Abs_{PBS}}{Abs_{H_{2}O}-Abs_{PBS}}\times 100\%$$

The average values of hemolysis and their standard deviations were calculated for n = 8 donors. Additionally, the samples after centrifugation were photographed.

### 2.4. Interaction with Human Serum

The interactions of nanocapsules containing CO, DADS or DATS with human serum were examined by dynamic light scattering (DLS) using a Malvern Zetasizer Nano ZS instrument (Malvern Instruments, Malvern, UK) working at 173° detection angle; measurements were performed at 22 °C. We monitored sizes of capsules, components of serum and eventual changes of sizes due to the interaction or coexistence of nanocapsules and fractions of serum components.

The non-anticoagulated blood was obtained from 9 healthy donors (aged 20–60 years) and centrifuged 2 or 3 times (10 min, 2000× *g*, Sigma 2–16K, Polygen, Wrocław, Poland) to extract human serum.

Nanocapsules were mixed with a serum 1:1 (*v*/*v*) and shaken using a vortex shaker (IKA, Königswinter, Germany). DLS measurements were run shortly after mixing and after 1 h of incubation at 37 °C. General purpose mode was used as a size distribution analysis algorithm and the reported data represents the mean values from 9 donors, each analyzed for four series of measurements (6 runs each), and their standard deviations (mean ± SD).

### 2.5. Interaction with Simulated Human Body Fluids

The interaction of nanocapsules containing CO, DADS and DATS with simulated human body fluids was examined by DLS. This experiment allowed us to observe the influence of low pH (in simulated gastric juice) and interaction with proteins/peptides (pepsin, amylase, lipase) on capsules. Simulated human body fluids were prepared as follows: simulated gastric juice (SGJ), composition: 0.2% (*w*/*v*), sodium chloride (Chempur, Piekary Slaskie, Poland), and 0.32% (*w*/*v*) pepsin (Sigma-Aldrich, Poznan, Poland) in 0.7% (*v*/*v*) hydrochloric acid (35–38%, Avantor Performance Materials Poland S.A., Gliwice, Poland, pH = 1.8), solution of amylase (0.1 L in PBS, pH = 7.4, Sigma-Aldrich, Poznan, Poland) and lipase (0.1 g/L in PBS, pH = 7.4, Sigma-Aldrich, Poznan, Poland).

Nanocapsules were mixed with the appropriate simulated body fluid in 1:1 ratio (*v*/*v*) and shaken using a vortex shaker (IKA, Königswinter, Germany). DLS measurements were run afresh after mixing and after 1 h of incubation at 37 °C. General purpose mode was used as a size distribution analysis algorithm and the reported data represents the averages from four series of measurements (6 runs each) and their standard deviations (mean ± SD).

### 2.6. Cell Culture

*Mus musculus* mammary gland animal stage IV human breast cancer cell line (4T1, ATCC:CRL-2539, Manassas, VA, USA) was grown in Petri dishes in Dulbecco’s Modified Eagle Medium (DMEM, high-glucose, Sigma-Aldrich, Poznan, Poland) supplemented with 5% (*v*/*v*) fetal bovine serum (FBS, HyClone, Warsaw, Poland) and 1% (*v*/*v*) penicillin-streptomycin solution (HyClone, Warsaw, Poland), at 37 °C in a humidified atmosphere containing 5% (*v*/*v*) of CO_2_. Cells reaching ~80% of confluence were passaged using trypsin solution (0.25%, 0.53 mM ethylenediaminetetraacetic acid (EDTA), HyClone, Warsaw, Poland). Before seeding cells for experiments, they were counted using Bürker chamber (Sigma-Aldrich, Poznan, Poland). A detailed information of the number of cells can be found in the descriptions of individual experiments.

For in vitro experiments samples of capsules were diluted in PBS, thus in the following descriptions there is an additional information about the concentration of the enclosed oil or the active compound in capsules (capsCO, capsDADS or capsDATS). For these experiments the dose of studied compounds is meaningful. In further experiments, described below in the Section 2.10, Section 2.11, Section 2.12, Section 2.13, Section 2.14 and Section 2.15, cells were incubated with appropriate formulations for 24 h, trypsinized and rinsed with PBS. The cells were then processed according to the procedure described below.

### 2.7. Cytotoxicity

The toxicity of nanocapsules with CO and mixtures of DADS and DATS with CO was tested on 4T1 cell line. The cells, 6 × 10^4^ cells per well, were seeded in 24-well plates and grown for 24 h. After that, cells were treated with appropriate formulations, i.e., capsCO, capsDADS, capsDATS and incubated for 24 h. The initial dispersions of the nanocapsules were diluted in PBS to vary the doses and the final concentrations of the capsules’ components (CO, DADS or DATS) that were calculated based on the amounts of the liquid in wells. Next, the cells were rinsed with warm DMEM solution and the neutral red uptake (NRU, Sigma-Aldrich, Poznan, Poland) or XTT (the cell proliferation kit II, Roche, Basel, Switzerland) assays were performed according to the manufacturer’s instructions with slight modifications (see details in [38]). The absorbance was measured using a microplate reader (Epoch2 BioTek, Winooski, VT, USA) at 540 and 700 nm for NRU, 460 and 700 nm for XTT assay. The cell viability was calculated as a percentage relative to the untreated control. Each value represents a mean of the three repetitions and the error bar is a standard deviation.

### 2.8. Scratch Assay

To investigate the influence of studied compounds on cancer cells migration, the scratch assay was conducted. Cells were seeded onto 24-well plate (3 × 10^4^ per well, a half less comparing to cytotoxicity assay as the final incubation in this experiment was 24 h longer) in 5% FBS in DMEM. After 24 h, cells were treated with PBS or nanocapsules (i.e., 28 mg/L CO in capsCO, 17 mg/L DADS in capsDADS and 8.5 mg/L DATS in capsDATS; non-toxic doses selected considering incubation in low content of FBS (1% *v*/*v*)). After another 24 h, the cell monolayer was scratched in each well. The medium was replaced with the fresh DMEM with 1% FBS to stop cell proliferation and to observe only a migration. Then, wells were treated again with appropriate nanocapsule formulation (the same amounts as previously) or PBS (control group). Wells were monitored by optical microscopy (Nikon Eclipse T2 with an objective Nikon 10×/0.25 and a camera DLT-Cam PRO 1.3 MP, Nikon, Tokyo, Japan) and microphotographs were made at 0 and 24 h after scratching and second capsules’ treatment.

### 2.9. Isolation of Total RNA

Total RNA was extracted from the cells using TRI reagent (Sigma-Aldrich, Poznan, Poland), according to the protocol provided by the manufacturer. The extracted RNA was suspended in ribonuclease free-water (Thermo Fisher Scientific, Waltham, MA, USA) and quantified by measuring of the absorbance at 260 nm (Genesys 10UV Scanning ultraviolet (UV)/Visible Spectrophotometer, Thermo Fisher Scientific, Waltham, MA, USA). After the procedure, the purity of the obtained RNA was determined by the spectrophotometric analysis (A_260nm_/A_280nm_). The integrity of the achieved RNA was confirmed by the separation of the 28S and 18S rRNA bands in 2.0% agarose-gel electrophoresis. Until further studies, the RNA solutions were stored at −80 °C.

### 2.10. Reverse Transcription of RNA

Total RNA from the cell samples was reverse-transcribed using a GoScript^TM^ Reverse Transcriptase Kit according to the manufacturer’s protocol (Promega, Madison, WI, USA). For a reverse transcription reaction, 3 μg of total RNA was mixed with 1 μL of Oligo (dT)_15_ primer (0.5 μg/reaction, Thermo Fisher Scientific, Waltham, MA, USA) and water pretreated with diethylpyrocarbonate (DEPC-H_2_O, Thermo Fisher Scientific, Waltham, MA, USA) and incubated for 5 min at 70 °C. After preincubation, other components were added to this mixture: 4 μL of GoScriptTM 5× concentrated reaction buffer (Promega, Madison, WI, USA), 3 μL of MgCl_2_ (final concentration 1.5–5 mM, Promega, Madison, WI, USA), 1 μL deoxyribonucleotide triphosphates (dNTPs, 10 mM, Thermo Fisher Scientific, Waltham, MA, USA), 1 μL of RNase inhibitor (20 U/μL, Thermo Fisher Scientific, Waltham, MA, USA) and 1 μL of GoScript^TM^ reverse transcriptase (Promega, Madison, WI, USA) (160 U/μL) in a total volume of 20 μL. The mixture was first incubated for 5 min at 25 °C, then for 60 min at 42 °C, and finally for 15 min at 70 °C. If necessary, the solutions of complementary DNA (cDNA) were stored at −20 °C.

### 2.11. Sulfurtransferases Gene Expression Measurement

The expression of the following genes (MPST, TST, CTH) was analyzed by polymerase chain reaction (PCR). As a reference (an internal standard), gene encoding glyceraldehyde 3-phosphate dehydrogenase (GAPDH, gene expressed normally in cells) was used. Amplification of cDNA samples was performed in a 25 μL reaction volume containing 2 μL of synthesized cDNA, 10 μM of each of gene-specific primer pairs (Table 1), 2 U/μL Taq DNA polymerase in 10 mM buffer Tris-HCl, pH 8.8 (Thermo Fisher Scientific, Waltham, MA, USA), 10 mM of each dNTPs and DEPC-H_2_O. In each case, a similar reaction was also performed in the mixture without DNA (the negative control) in order to confirm the specificity of the obtained reaction products.

The temperature profile of PCR amplification for these genes consisted of Taq polymerase activation at 94 °C for 5 min, denaturation of cDNA at 94 °C for 30 s, primer annealing at 58 °C—TST, 59 °C—GAPDH, 60 °C—CTH, 62 °C—MPST, for 30 s, elongation at 72 °C for 2 min for the following duration: 28 cycles for GAPDH, 29 cycles for TST, 30 cycles for CTH and MPST, and was completed by the extension step for 8 min. All the mRNA sequences of the tested genes were obtained from the National Center for Biotechnology Information (NCBI, Bethesda, MD, USA) and all the primer sequences were synthesized by the DNA Sequencing and Synthesis Service—Institute of Biochemistry and Biophysics Polish Academy of Sciences (IBB PAN) in Warsaw, Poland. The PCR conditions for these four genes were established and optimized specifically to address the needs of the present study, they are published for the first time in this paper. All the amplification reactions were performed at least three times to ensure the accuracy of the results. All the PCR products were analyzed by electrophoresis on 2.0% agarose (Sigma-Aldrich, Poznan, Poland) gel stained with ethidium bromide (Sigma-Aldrich, Poznan, Poland), directly visualized under a UV light and photographed (ChemiDocTM MP Imaging System with Image Lab Software, version 6.0, Bio-Rad, Hercules, CA, USA).

### 2.12. Cell Homogenization

The 4T1 cells (3.5–5 × 10^6^ cells) were suspended in 0.1 M phosphate buffer (pH = 7.5), in the proportion of 1 × 10^6^ cells/0.04 mL of the buffer and sonicated 3 × 5 s at 4 °C (Bandelin Sonoplus GM 70, Bandelin electronic GmbH and Co. KG, Berlin, Germany). After centrifugation (1600× *g* for 10 min, MPW-260R, MPW MED. INSTRUMENTS, Warsaw, Poland) the supernatant was used to determine the protein concentration, sulfane sulfur content and the activity of rhodanese (TST, EC 2.8.1.1), 3-mercaptopyruvate sulfurtransferase (MPST, EC 2.8.1.2) and cystathionine γ-lyase (CTH, EC 4.4.1.1). For the determination of low molecular weight sulfur-containing compounds using reversed-phase high-performance liquid chromatography (RP-HPLC), by the procedure described below in point 2.14, the pellets of cells were suspended in 250 μL mixture containing 0.9% NaCl (Sigma-Aldrich, Poznan, Poland)/1 mM bathophenanthrolinedisulfonic acid disodium salt (Sigma-Aldrich, Poznan, Poland)/70% perchloric acid (PCA, Sigma-Aldrich, Poznan, Poland), sonicated 3 × 5 s at 4 °C. The sediment was separated by centrifugation (1600× *g* for 10 min, MPW-260R, MPW MED. INSTRUMENTS, Warsaw, Poland) and the supernatant was saved at −80 °C until used for the RP-HPLC analyses.

### 2.13. Enzyme Assays

#### 2.13.1. Rhodanese (TST) Activity

TST activity was assayed by the Sörbo’s method [42], following a procedure described by Wróbel and others [43]. The incubation mixture contained: 200 μL of 0.125 M sodium thiosulfate (Sigma-Aldrich, Poznan, Poland), 100 μL of 0.2 M potassium dihydrogen phosphate (Sigma-Aldrich, Poznan, Poland), 100 μL of homogenates, 100 μL of 38% formaldehyde (Avantor Performance Materials Poland S.A., Gliwice, Poland) (only blank sample) and 100 μL of 0.25 M potassium cyanide (Merck, Darmstadt, Germany). The mixture was incubated for 5 min at room temperature. Subsequently, 100 μL of 38% formaldehyde (only tested sample) and 500 μL of 0.2 M ferric nitrate reagent (Sigma-Aldrich, Poznan, Poland) were added to all samples. The amount of thiocyanate formed during the reaction catalyzed by TST was measured colorimetrically at 460 nm (Genesys 10UV Scanning UV/Visible Spectrophotometer, Thermo Scientific, Waltham, MA, USA). The enzyme units were defined as nmoles of SCN^−^, which are formed during 1 min incubation per 1 mg protein.

#### 2.13.2. 3-Mercaptopyruvate Sulfurtransferase (MPST) Activity

An MPST activity was assayed according to the method of Valentine and Frankenfeld [44]. The incubation mixture contained: 250 μL of 0.12 M sodium phosphate buffer, pH 8.0, 50 μL of 0.5 M sodium sulfate (Sigma-Aldrich, Poznan, Poland), 50 μL of 0.15 M D, L-dithiothreitol (DTT, Sigma-Aldrich, Poznan, Poland), 50 μL of distilled water, 50 μL of supernatant and 50 μL of 0.1 M 3-mercaptopyruvate acid sodium salt (Sigma-Aldrich, Poznan, Poland) of the final volume of 500 μL. The mixture was incubated for 15 min, then 250 μL of 1.2 M PCA was added to stop the reaction. Samples were centrifuged at 1600× *g* for 5 min, and then 100 μL of supernatant was transferred to a 1350 μL solution containing: 1200 μL of 0.12 M sodium phosphate buffer, pH 8.0, 100 μL of 0.1 M N-ethylmaleimide (NEM, Sigma-Aldrich, Poznan, Poland) and 50 μL of β-Nicotinamide adenine dinucleotide reduced disodium salt hydrate (NADH, Sigma-Aldrich, Poznan, Poland) (5 mg/mL). After equilibration at 37 °C, 2.5 μL (7 IU) of L-lactate dehydrogenase (LDH, Sigma-Aldrich, Poznan, Poland) was added, and the decrease in absorbance was measured at 340 nm (Genesys 10UV Scanning UV/Visible Spectrophotometer, Thermo Fisher Scientific, Waltham, MA, USA). The MPST activity was expressed as nmoles of pyruvate produced during one minute-incubation at 37 °C per 1 mg of protein.

#### 2.13.3. Cystathionine γ-Lyase (CTH) Activity

A CTH activity was determined by Matsue and Greenberg’s method [45] with some modifications described by Czubak et al. [46]. The incubation mixture contained: 25 μL of 1.3 mM pyridoxal phosphate (Sigma-Aldrich, Poznan, Poland), 25 μL of 0.02 mM EDTA (Sigma-Aldrich, Poznan, Poland), 250 μL of 45 mM cystathionine (Sigma-Aldrich, Poznan, Poland) in 0.1 M phosphate buffer, pH 7.5 (2.5 mg cystathionine per sample), and 75 μL of supernatant. The 0.1 M phosphate buffer, pH 7.5, containing 0.05 mM 2-mercaptoethanol (Fluka Chemie GmbH, Buchs, Switzerland) was added to obtain a final sample volume of 650 μL. The reaction was stopped after 30 min of incubation at 37 °C by placing 125 μL of incubation mixture in 25 μL of 1.2 M PCA. Samples were centrifuged (1600× *g* for 10 min, MPW-375, MPW MED. INSTRUMENTS, Warsaw, Poland) and 25 μL of supernatant was transferred to 625 μL of 0.194 mM NADH and kept at 37 °C. Control samples (without 45 mM cystathionine) were prepared in the same way as the examined samples. The measurement (absorbance at 340 nm; Genesys 10UV Scanning UV/Visible Spectrophotometer, Thermo Fisher Scientific, Waltham, MA, USA) was conducted through 180 s, while after 10 s, 25 μL (9.06 IU) of LDH was added. The difference between the initial volume of absorbance (before LDH addition) and the lowest value (after LDH addition) corresponded to the amount of α-ketobutyrate formed in the course of the cystathionase reaction. The CTH activity was expressed as nmoles of α-ketobutyrate formed during one-minute-incubation at 37 °C per 1 mg of protein.

#### 2.13.4. Sulfane Sulfur

Sulfane sulfur level was determined by the method of Wood [47]. This method is based on a cyanolysis reaction and colorimetric determination of ferric thiocyanate complex ion. Incubation mixtures in a final volume 880 μL contained: 20 μL 1 M ammonia solution (Avantor Performance Materials Poland S.A., Gliwice, Poland), 20 μL homogenate, 740 μL H_2_O, and 100 μL 0.5 M potassium cyanide (Merck, Darmstadt, Germany). The incubation was performed for 45 min at room temperature. After incubation, thiocyanate was estimated colorimetrically at 460 nm (Genesys 10UV Scanning UV/Visible Spectrophotometer, Thermo Fisher Scientific, Waltham, MA, USA) after addition of 20 μL 38% formaldehyde (Avantor Performance Materials Poland S.A., Gliwice, Poland) and 40 μL ferric nitrate reagent (Sigma-Aldrich, Poznan, Poland). The level of sulfane sulfur was expressed as nmoles of SCN^-^ (thiocyanate) per 1 mg of protein.

#### 2.13.5. Protein Content

Total protein content was determined by the method of Lowry and others [48]. The crystalline bovine serum albumin (BioShop Canada Inc. Burlington, ON, Canada) was used as a standard.

### 2.14. Determination of Concentrations of Low Molecular Weight Sulfur-Containing Compounds using Reversed-Phase High-Performance Liquid Chromatography (RP-HPLC)

The RP-HPLC method of Dominick et al. [49] with the modifications described by Bronowicka-Adamska et al. [50] was used to determine the level of low molecular weight sulfur-containing compounds, such as reduced (GSH) and oxidized (GSSG) glutathione, cysteine and cystine (the chromatographic system consisted of LC-10 Atvp Shimadzu Corp. (Kyoto, Japan) pumps, four channel degassers, column oven and a Shimadzu Corp. SIL-10 Advp autosampler; the chromatographic peaks were measured by a Shimadzu Corporation SPD-M10Avp-diode array detector; LabSolution (Warsaw, Poland) LC software was used to control system operation and facilitate data collection). The standard curves were generated in the supernatant obtained from cellular homogenates in the range from 13 to 75 nM of each compound per mL. All the standard curves generated for the analyte were linear in the investigated concentration range.

### 2.15. Statistical Analysis

The results were presented as means ± standard deviation (SD).

The significance of the differences between controls and examined groups were calculated depending on whether the data meet the assumptions of normality and variance homogeneity: either using Student’s *t*-test or the Mann–Whitney U test. For more than two groups analysis of variance (ANOVA) or the non-parametric Kruskal–Wallis test (Section 3.3.1) were used, with the post-hoc multiple comparison by Tukey test or the Conover-Inman’s test, respectively.

For the experiments of interactions of capsules with human serum (Section 3.3.2) the normal distribution of data was verified by the Shapiro–Wilks test. The statistical significance of differences was estimated with a two-tailed Mann–Whitney U test for data without normal distribution or with unpaired Student’s *t*-test for data meeting the criteria of normal distribution. Statistical analyses were performed using Statistica v.13 (Dell Inc., Tulsa, OH, USA).

For the experiments of interactions of capsules with simulated body fluids and cytotoxity (Section 3.3.3 and Section 3.4.1) the significance of the differences between diameter values was calculated with the Mann–Whitney U test two-group comparisons (in experiments with simulated body fluids in relation to the respective control groups: capsCO, capsDADS and capsDATS with PBS at the time point ‘0 h’, and in cytotoxicity experiments in relation to the respective control groups) and a probability value (*p*-value) of less than 0.05 was considered to be significant.

All experiments described in the Section 3.4.3 and Section 3.4.4 were repeated at least three times. Statistical analyses were performed using the Statistica software, version 13.3 (TIBCO, Software Inc., Palo Alto, CA, USA).

## 3. Results and Discussions

### 3.1. Nanocapsules as Delivery Systems of Hydrophobic Compounds

The studied systems, HA-based nanocapsules, belong to self-assembled oil-core type carriers [19,37,38]. Self-organization is realized by hydrophobically modified HA which acts as the shell and the stabilizer through the immersion of the hydrophobic alkyl chains inside the oil core. Advantageously, no low molecular surfactants, which may exhibit thermodynamic instability during dilution, undesirable in systems for potential in vivo applications, are needed. Furthermore, the shell is a barrier against potential damaging factors for encapsulated compounds, e.g., oxidizing, or hydrolyzing agents, as well as a protective layer for the outside environment against the premature action of encapsulated actives or side effects, including aggregation in blood vessels. The shell may also affect the circulation time in the bloodstream. Moreover, HA specifically binds to a group of receptors, e.g., CD44, RHAMM, enabling the targeted therapy [51,52].

The oil-core acts as the microenvironment for hydrophobic active substances, herein the garlic oil-originated DADS and DATS. The continuous phase is a corn oil, used as an inert vehicle possessing relatively high oxidation stability, high technological and nutritional qualities, and exhibiting potential in the food and biomedical industries.

**Figure 2:**
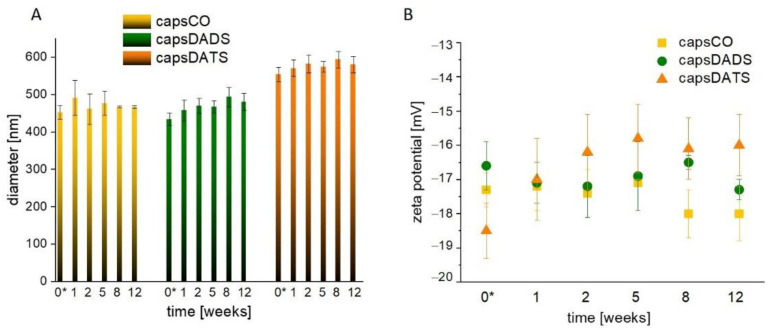
Time changes of averaged volume-weighted diameters (**A**) and averaged zeta potentials (**B**) of nanocapsules. Data presented as mean +/− standard deviation (SD); n = 4. Observations were conducted for 12 weeks. *—measurement at the day of capsules preparation.

The nanocapsules are obtained based on the previously described, reproducible emulsification method [53]. However, due to a different compositions of the cores, we monitored the changes in sizes and zeta potentials of capsules over the few weeks to control their stability. In the Figure 2A comparable hydrodynamic diameters of capsCO and capsDADS (approximately 480 nm) are presented while capsDATS are characterized by larger hydrodynamic diameters, approximately 560 nm. The sizes of capsCO are only slightly varying during the first weeks of observations and then their diameters become very stable. Similarly, the sizes of capsDADS and capsDATS do not change significantly after small variation during the first 2 weeks. The observed differences in the nanocapsules’ sizes are likely caused by differences in densities of CO (~0.9 g/mL), DADS (~0.9 g/mL) and DATS (~1.1 g/mL). The density of the core may affect the physicochemistry of the obtained capsules, including the efficiency of the emulsification process as well as the Brownian motion measured using the DLS technique. Furthermore, regarding the DATS density value is greater than water density, the oil may slowly sediment in oil-water solution. The fluctuations in diameters during the 12 weeks of observations were caused by achieving the thermodynamic stability in various conditions, as the nanocapsules were stored at ~4 °C, while the measurement was run at 22 °C. Moreover, the variations may occur due to the reorganization and disintegration of some aggregates. However, after the first two weeks, relative stability, given as the lack of size changes, was achieved. The diameter characteristics were supported by zeta potential observations (Figure 2B) showing similar dynamics: the small changes during the first days leading to long-term stability. Importantly, the zeta potential value varied only in the range from approximately −16 to −19 mV. Despite the fact that values of zeta potentials did not meet the electrostatic stability conditions of colloids (the absolute value of zeta potential >30 mV), they exhibited only small variations in time that did not affect the overall stability of the studied emulsions provided also by the steric repulsion between the shell-forming polymers.

### 3.2. Stability of DADS and DATS in Oxidizing Conditions

Organic, biologically active molecules including drugs and pharmaceuticals are susceptible to chemical decomposition, through the interaction with proteins, reactions with enzymes or through the inappropriate use and/or storage, which often contributes to the loss of their therapeutic potential. The main chemical reactions affecting the stability of compounds are oxidation and hydrolysis initiated by the presence of O_2_, water, light, heat, or certain trace metals.

**Figure 3:**
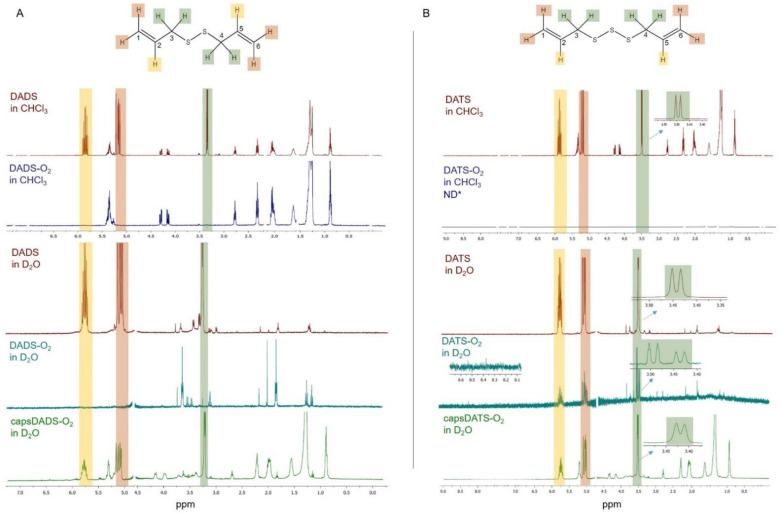
Nuclear magnetic resonance (^1^H NMR) spectra of native DADS, after oxidation (DADS-O_2_) and the encapsulated DADS after oxidation (capsDADS-O_2_) measured either in CHCl_3_ -d or D_2_O (panel (**A**)). Analogous data for DATS are presented in panel (**B**). Signals from solvents were cut out as described in Methods. * ND, signals not detected.

In oxidizing conditions, DADS and DATS become oxidized and decompose. The chemical disintegration may be realized by attaching oxygen atoms to sulfur atoms or through the oxidation of double alkyl bonds. We monitored the destabilization under oxidizing conditions using ^1^H NMR spectroscopy (Figure 3). The nanocapsules for these experiments were prepared in D_2_O (replacing PBS) while the spectra of non-encapsulated DADS and DATS before and after oxidation were analyzed in both D_2_O and CHCl_3_-d to dissolve the oxidation products with various polarities. The spectra of the native compounds show the characteristic signals of all hydrogens both in D_2_O and CHCl_3_-d (Figure 3, signals indicated by appropriate colors). Briefly, signals in the range of ~5.7–5.9 ppm are assigned to hydrogen atoms at C2 and C5 (see compounds structures in Figure 3), those at ~5.0–5.2 ppm to hydrogens at C1 and C6, and those at ~3.3–3.5 ppm to hydrogens at C3 and C4.

The spectra of non-encapsulated DADS and DATS changed significantly after oxidation. While the signals of the starting compounds (well visible in the spectra in CHCl_3_-d) diminished, a few new signals appeared, which can be assigned to the possible products of oxidation-degradation caused by O_2_. The results suggest that the oxidation process leads to the formation of a mixture of products, thus, an unambiguous assignment of all the peaks may be challenging. Interestingly, comparing the spectra of the compounds before and after oxidation, we can see that the native DADS and DATS are already partially decomposed (during storage) as we can observe some extra signals assigned to the oxidation products that become dominant after oxidation. Nevertheless, some of them may also originate from impurities in original compounds.

The main change observed in the spectra of DADS-O_2_ in D_2_O and CHCl_3_-d (Figure 3A) is the disappearance of signals at ~5.0–5.2 ppm and ~5.7–5.9 ppm that is caused by the rearrangement of double alkyl bonds into single bonds, and the formation of, e.g., hydroxyl or carbonyl groups. However, the signals from aldehyde or carboxyl groups are not visible, which may suggest their absence or low content (with a signal being below the detection limit). Rearrangement of the signals at ~3.2–3.6 ppm indicates the presence of asymmetric structures with at least one oxidized sulfur atom. This also applies to the unoxidized sample.

In spite of DADS and DATS being similar compounds, the impact of O_2_ is different. First of all, the double bonds present in DATS seem to be stronger than in DADS, as even after twice longer oxidation the initial signals from these double bonds (~5.1–5.2 ppm and ~5.7–5.9 ppm) are detectable indicating that some molecules still have alkyl groups. Interestingly, Yoo et al. [34] reported better stability of DATS than DADS under storage at 35 °C during 3 months that is in agreement with the results obtained here. However, the signal from hydrogens at C1 and C6 changed shape due to the change of chemical environment (oxidation of at least one sulfur atom or presence of some hydroxyl or carbonyl groups nearby). Secondly, the products of DATS oxidation are detectable only in the spectrum measured in D_2_O as they seem to be insoluble in CHCl_3_-d (Figure 3B). A greater amount of sulfur atoms in DATS results in the larger number of products of sulfur oxidation. Thus, the mixture of products, including some asymmetric ones, results in the presence of additional doublets or singlets at 2.75 ppm, 2.95 ppm, and 3.4–3.6 ppm. The small signal from the hydrogen of an aldehyde group (or neighboring of carboxyl group) is detectable at ~8.4 ppm (Figure 3B).

Importantly, it was observed that applying oxidation procedure to the capsule samples does not totally destroy the encapsulated DADS and DATS as the signals were, to large extent, preserved. Thus, it may be concluded that HA-based shell of capsules acts as a barrier for O_2_ and protects the components of the cores against oxidizing conditions, significantly slowing down the process. This conclusion makes the presented delivery systems promising candidates for oily, hydrophobic compounds exhibiting instability in oxidizing conditions, like the studied DADS and DATS.

### 3.3. Interaction with Blood and Digestive Track Components

Numerous DDSs were found to interact with various blood components causing hemolysis, blood aggregation or agglutination, among others, and with digestive tract proteins, enzymes or under specific conditions (i.e., low pH), which may also cause toxic effects, digestion, aggregation, or secretion of proinflammatory agents [54,55]. Thus, we decided to investigate how the hyaluronate-based nanocapsules with encapsulated garlic oil components interact with various body fluids.

#### 3.3.1. Hemolysis

The disintegration of red blood cell (RBC) membrane is one of the major barrier to in vivo applications of DDSs or medicines [28]. Sulfides are of special interest due to observations that garlic and onion cause oxidative hemolysis, leading to anemia, which was proven in a study considering domestic and laboratory animals [56].

**Figure 4:**
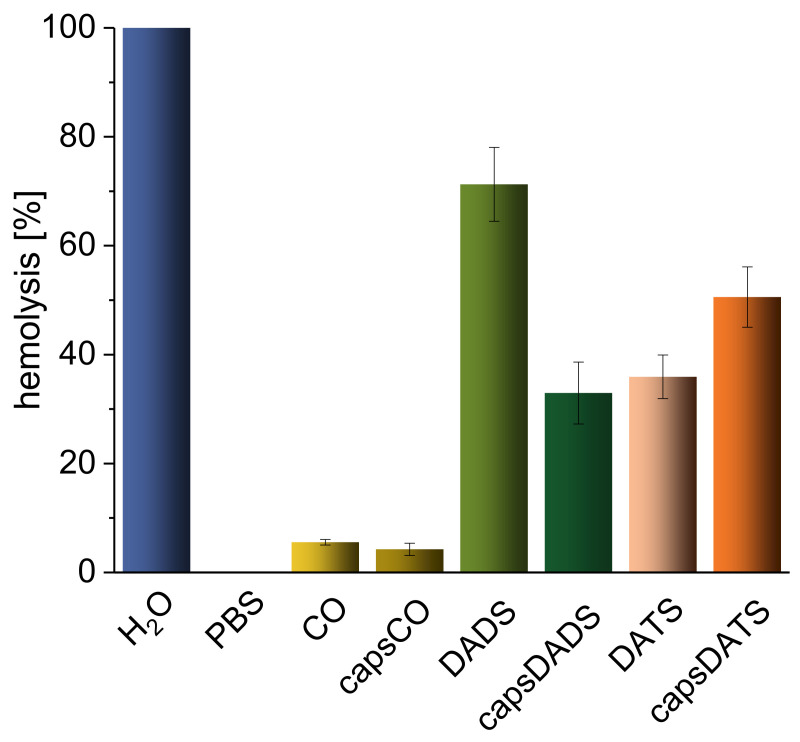
Hemolytic effects of tested emulsions: capsCO, capsDADS, capsDATS and free oils. Results are presented as mean +/− standard deviation (SD); n = 4–8 repetitions. Statistical significance, estimated with non-parametric one-tailed Kruskal–Wallis test and *post hoc* non-parametric Conover–Inman’s test, was as follows: all samples > phosphate-buffered saline (PBS), *p*_1,α_ < 0.0001; CO = capsCO, n.s.; capsDADS > capsCO, *p*_1,α_ < 0.0001; capsDATS > capsCO, *p*_1,α_ < 0.0001; DADS > capsDADS, *p*_1,α_ < 0.0001; DATS < capsDATS, *p*_1,α_ < 0.01.

Herein, we compare the effect of encapsulated and free DADS and DATS on the integrity of erythrocyte membranes by in vitro hemolysis assay. The factor enabling the assessment of toxicity was the absorbance of hemoglobin released from erythrocytes (RBCs) (Figure 4). Following the procedure, the positive control (deionized water) caused the complete hemolysis due to osmotic pressure while the negative control (PBS) did not affect the membrane integrity (Figure S1). “Empty” capsules (without garlic oil components) and CO alone (capsCO and CO, respectively) exhibited the hemolytic effect in the range of 5%, which is considered very low or biologically not significant impact (according to literature reports [36,57]). After incubation with DADS, 80% of RBCs were lysed, while for capsDADS we observed two-fold decrease of hemolysis. The hemolytic effect of capsDATS and DATS was at the similar level (approximately 45%, taking into consideration their standard deviations) (Figure 4). This protective anti-hemolytic effect of encapsulation of DADS seems to be important from a biological point of view since only free DADS induced hemolysis at the level of positive control (H_2_O). The pictures in Figure S1 support the obtained results, showing released hemoglobin from destructed erythrocytes. Moreover, in each tube except the positive control, the RBC precipitate is noticeable.

Herein, we preliminarily confirmed the protective function of used polysaccharide-based capsules. Although the value of hemolysis of encapsulated DADS is still quite high, the examined doses in in vivo conditions are prone to be more diluted after delivery into the organism, lowering the effect. It may be studied further in subsequent research, including in vivo testing.

On the other hand, for DATS, no significant difference in the hemolytic effect between encapsulated and non-encapsulated drug can be observed. This divergence in the results can be explained when the physicochemical differences between studied compounds is taken into consideration—DATS has higher density than DADS, when comparing both to water. Hence, DATS and capsules with enclosed DATS may be not well mixed and form non-homogenous suspension/mixture which may explain the observation presented in Figure 4 and Figure S1. However, further investigations considering interactions with other blood components should provide more complementary information. Moreover, as mentioned, the concentrations of compounds of interest in blood after potential administration into living organisms would be smaller than in in vitro experiments. This leads to a conclusion that the emulsions obtained could be relatively safe carriers of DADS and DATS in the circulation.

#### 3.3.2. Interaction with Human Serum

**Figure 5:**
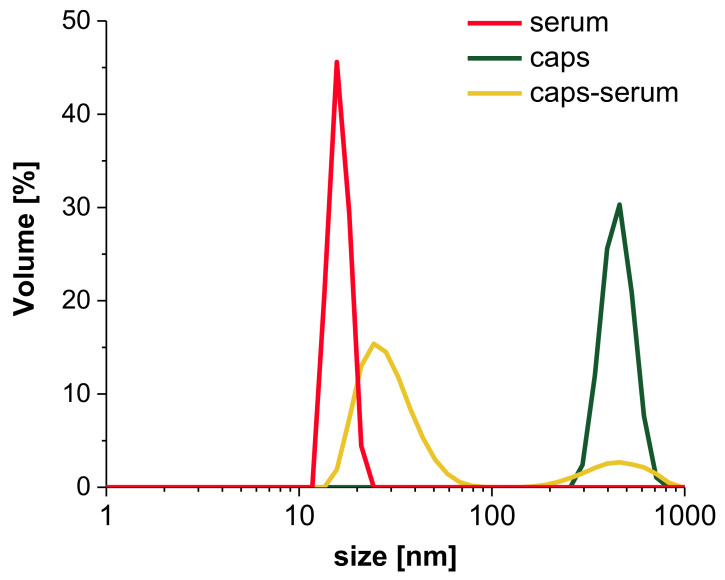
Representative volume-weighted particle size distributions of serum, the capsules and capsules interacting with serum, determined by dynamic light scattering (DLS) measurements.

For further analysis of interaction of capsules with non-morphotic blood components, the human serum was investigated. The samples were incubated with either human serum collected from 9 healthy donors or PBS. The volume-weighted size distributions of capsules (Figure 5) and averaged diameters (Table S1) were analyzed. The analysis of Figure 5 indicates, that serum components did not exceed 20–40 nm in diameter, which is associated with the sizes of proteins and antigens present in the sample (red line). The size of capsules (green line) reaches approximately 450 nm (Figure 5). When the capsules were mixed with serum, the particle size distribution is dominated by small objects, and the fraction of bigger objects, which scatters the light to the greatest extent, is the smallest by volume. The increase in dispersity (PdI) and standard deviation values, and the decrease in the average diameters observed in the sample containing capsules in serum indicate numerically the concomitance of the two fractions. Those values are not physically significant but with complementary size distribution observations, the results rather suggest the coexistence of serum and capsules fractions with no additional bigger aggregates resulting from the interactions between the two fractions’ constituents.

A list of average sizes of samples presented in Table S1 supports the observations made based on the particle size distributions (Figure 5). The coexistence of serum components and capsules is also visible here. The fraction of small serum components, with the objects ranging between 11 nm and 48 nm, dominates the size results of all samples. Additionally, we observed the growing dispersity of samples containing serum, compared with the capsules suspended in PBS, given by approximately two- to-three fold increase in PdI value. Moreover, the observations let us postulate that the time of incubation does not significantly affect the interaction between serum and capsules. Thus, we presented herein the results obtained for samples after 1 h incubation at 37 °C.

#### 3.3.3. Interactions with Digestive Track’s Proteins and Influence of Low pH

**Figure 6:**
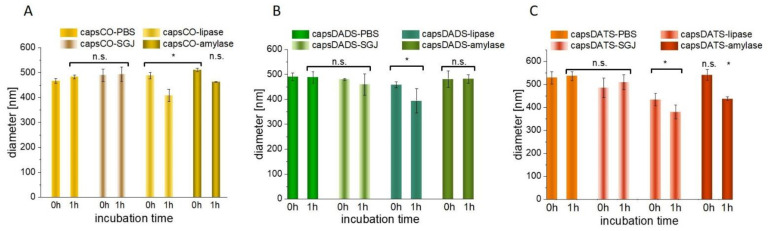
Interaction of capsCO (**A**), capsDADS (**B**) and capsDATS (**C**) with a simulated gastric juice (SGJ), lipase and amylase solution. The data shown as mean +/− SD, (n = 4) represents the diameters of capsules in freshly prepared samples and after 1 h incubation at 37 °C. Significance estimated with the Mann-Whitney U-test in relation to the respective control groups; * *p* < 0.05; n.s.—not significant.

The widely described issue dealing with the interactions of polysaccharides and peptides [58,59], also covers the problems with their undesirable mutual influences. Among numerous literature examples, the promising chitosan-alginate-based capsules masking unpleasant shark liver oil taste were disrupted by enzymes lipase or pancreatin, among others [60]. To investigate how oral administration can affect the properties of HA-based delivery system, we examined the properties of nanocapsules in digestive enzyme solutions: amylase and lipase (neutral pH) and also in simulated gastric juice (SGJ), which is characterized by low pH (1.8). The minor changes in the size of capsules incubated with SGJ were found to be insignificant (Figure 6), which remains in agreement with our previous work describing the stability of nanocapsules with enclosed curcumin [61]. Each type of examined capsule interacted slightly with lipase, which was indicated by the decrease in size, with the effect being strengthened after 1 h incubation. Given that lipase is an enzyme which hydrolyzes lipids [62], and as the capsule CO core contains mostly a mixture of lipids and fatty acids, small reorganizations may be observed. The degradation may refers to any free CO, which has not been successfully enclosed in the capsule core. Although, those negligible amounts of free CO did not affect the overall capsule evaluation, they may be responsible for the observed lipase-mediated degradation resulting in decreasing size. The presence of hyaluronate shell shows the protective effect, which manifests in relatively small changes in size (Figure 6). Some variations were also observed for samples incubated with amylase (regarding capsCO and capsDATS), which may be caused by the enzyme property of cleavage alpha-1,4 linkages and degradation of many polysaccharides (Figure 6). Interestingly, the effect for HyC12 is not strong, which may be the result of its organization onto spherical structures and hydrophobic modification. Indeed, the HA modified with sulfhydryl ligand cysteine ethyl ester studied by Laffleur et al. [63] showed less sensitivity towards enzymatic amylase degradation in comparison with an unmodified one. The most visible effect for interaction of capsDATS after 1 h incubation is their smaller diameter in comparison with capsCO and capsDADS. However, this does not indicate capsule degradation.

Concluding, the observed changes in capsule diameters incubated with enzyme solutions were not significant from the perspective of the cargo protection. Due to the polysaccharide organization, spherical oil droplets are protected against enzymatic degradation and low pH environment.

### 3.4. Influence on Cancer Cells

#### 3.4.1. Cytotoxicity

The investigated garlic oil components, namely DADS and DATS, are consider to exhibit cytotoxic effects against various types of cancer. They follow different mechanisms of action including formation of DNA adducts, influencing cell cycle arrest, inducing apoptosis, triggering an antioxidant effect etc. [5]. Regarding the literature data, the cytotoxic effect of DADS and DATS is studied mainly for compounds dissolved in DMSO. In 2001, Nakagawa and coworkers [13] published a paper, in which toxicity and effect of DADS on four human breast cancer cell lines (KPL-1, MCF-7, MDA-MB-231 and MKL-F) were studied. The IC50 value (half maximal inhibitory concentration) of DADS delivered in DMSO was in the range of ~0.3–2.7 mg/L. Another research group studying the influence of DADS on human breast cancer cell line MCF-7, specified the concentration of DMSO to be 0.1% and showed approximately 50% cell viability at a dose of ~59 mg/L [14]. The same cell line treated by DATS in DMSO at a dose of approximately 9 mg/L resulted in ~40% cell viability, while DADS in DMSO at the dose of approximately 73 mg/L induced ~90% cell viability [15]. The 4T1 cell line treated with organosulfur compounds delivered as a mixture of n-propyl polysulfides resulted in IC50 ~44 mg/L [64].

Herein, we examined if encapsulated compounds still have the potential in triggering the anticancer effect on culture of mammary cancer 4T1 cell line. Our previous experiment showed that the release of encapsulated oils inside cells improved the anticancer activity of the compound as compared with its non-encapsulated counterpart [38]. The delivery of active compound inside the cores played a protective role against its degradation before reaching the site of action. After capsule incorporation, the hyaluronidases present inside the cells cut the polysaccharide shell, which led to drug release.

**Figure 7:**
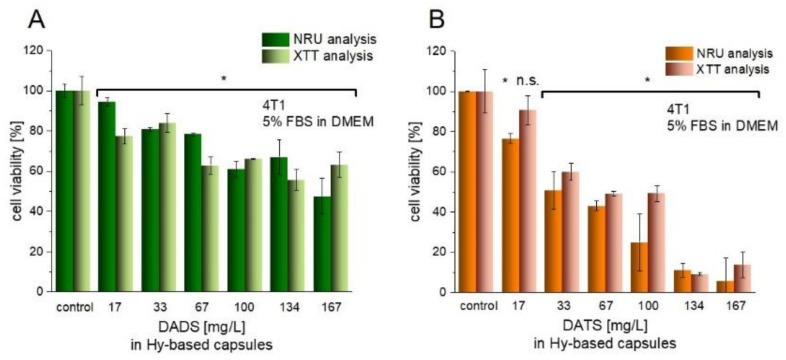
Cell viability (analyzed by neutral red uptake (NRU) and XTT assays) after the treatment of Hy-based capsules with DADS (**A**) and DATS (**B**). The data shown as mean +/− SD, (n = 3) represents the amount of encapsulated and delivered compounds. The aqueous and oil phase were mixed in 1000:3 volume ratio. Significance estimated with the Mann–Whitney U-test in relation to the respective control groups; * *p* < 0.05; n.s., not significant.

In this work, the cell viability upon treatment with hyaluronate-based nanocapsules was examined. The nanocapsules without active compounds (capsCO) did not exhibit the toxic effect on cultured cells (Figure S2), and the small changes were determined as not significant. Results obtained from two different cytotoxic tests, namely XTT and NRU, turned out to be complementary. In the case of nanocapsules containing different doses of either DADS or DATS encapsulated in oil cores, the toxic effect on cancer 4T1 cells was detected. Comparing the doses with other researchers (described above), one may observe that encapsulation of DADS and DATS in hyaluronate-based nanocapsules led to a decrease in anticancer activity. After 24 h of incubation, the cancer cell viability was lowered up to approximately 50% after the administration of 33 mg/L and 167 mg/L DATS and DADS in capsules, respectively (Figure 7). However, the enormous advantage represented by the replacement of unfavorable solvent into the inert carrier, opens the new way of potential therapy without reducing its applicability. Avoiding the use of DMSO eliminates its toxic effect on cells and excludes an additional source of sulfur, which can affect the investigated system in an uncontrolled manner.

#### 3.4.2. Cell Migration

**Figure 8:**
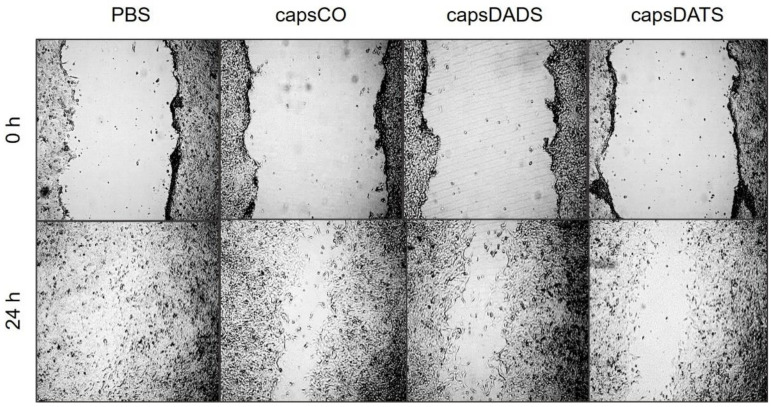
Representative microphotographs obtained from an optical microscope: 4T1 cells’ monolayer freshly after scratching (upper panel) and 24 h after the treatment with PBS, capsCO, capsDADS and capsDATS, respectively (lower panel). Images taken in the same sample positions.

Taking into consideration that the metastasis and angiogenesis are the most important aspects of the expansion of cancer, the migration process of neoplastic cells is critical. Therefore, the possibility of influencing cell migration would be beneficial for the formulations with proven toxicity against cancer cells. Due to the fact that the study of the biological processes driving cell migration is a complex and multidirectional task, which was not the direct goal of this work, we assessed the impact of the studied systems on cell migration only by performing a scratch assay. Although it is a simple and basic method, its effectiveness in assessing the influence of compounds on cell migration is well-documented in the literature [65,66,67,68]. The experiment we performed was based on the observation of the cell migration into the generated free space in the cell monolayer. The width of this space and the exposure time in the presence of the tested compounds and the control (PBS) were the same, which allows the assessment of inhibition in the movement of tumor cells. The data obtained show that the presence of the tested formulations reduces significantly the migration of cancer cells (Figure 8). In the case of the scratched monolayer of the control sample (PBS treated), cells migrated and closed the artificial wound. By contrast, after the treatment with capsules containing garlic oil components at very low doses, the clearance is visible after 24 h. Importantly, the effect is the strongest for the monolayer treated with capsDATS, which stays in agreement with the cytotoxicity results.

#### 3.4.3. Determination of Low Molecular Weight Sulfur-Containing Compounds

It was proven that garlic-derived DADS and DATS influenced the cysteine non-oxidative metabolism in mammalian cells [11,12]. Therefore, we investigated whether the encapsulation of DADS and DATS in hyaluronate-based nanocapsules affects their structure and biological function. The data shown in Table 2 indicate that the tested drug extracts encapsulated within the capsule cores increased the intracellular cysteine level, specifically by 4.4-fold for encapsulated DATS and above 5.6-fold for encapsulated DADS. Cysteine in the cells can be used in two ways: (1) for glutathione synthesis (because the level of reduced glutathione is maintained at the control level in the case of capsDATS, or it increases more than 2.5 times as it does after administration of capsDADS, Table 2), and/or (2) for L-cysteine desulfuration pathways (we observe statistically significant changes in the activity of selected sulfurtransferases using both formulations (Table 3). The treatment with capsDADS also results in an increase in oxidized (GSSG) and total glutathione levels (about 2.7-fold) in comparison to control (Table 2). However, this increase can be neglected because in both the control group and the test group, the percentage of oxidized glutathione calculated relative to reduced glutathione was about 4%. An increase in reduced glutathione entails an increase in oxidized glutathione, but their ratio remains the same, so this is not statistically significant even though the results obtained for oxidized glutathione under isolated conditions might suggest such finding.

The opposite situation can be observed when capsDATS formulation (both tested concentrations: 17 and 33 mg/L) is administered to cells. That leads to a decrease in oxidized glutathione level, with unchanged levels of reduced glutathione and increased amounts of cysteine (Table 2). Greater availability of cysteine increases the pool of locally available low molecular weight antioxidants such as glutathione which, due to the presence of the -SH group, prevents oxidation of other biologically important molecules. However, the reduction of GSSG levels in the group treated with capsDATS (33 mg/mL), directly implicates the result of the ratio of reduced to oxidized forms of glutathione, suggesting the presence of oxidative stress in these cells, which does not seem to be the truth. When the redox state in cells is maintained, the ratio [GSH]/[GSSG] is high [69].

#### 3.4.4. Sulfane Sulfur Level and Sulfurtransferases Activity and Expression

**Figure 9:**
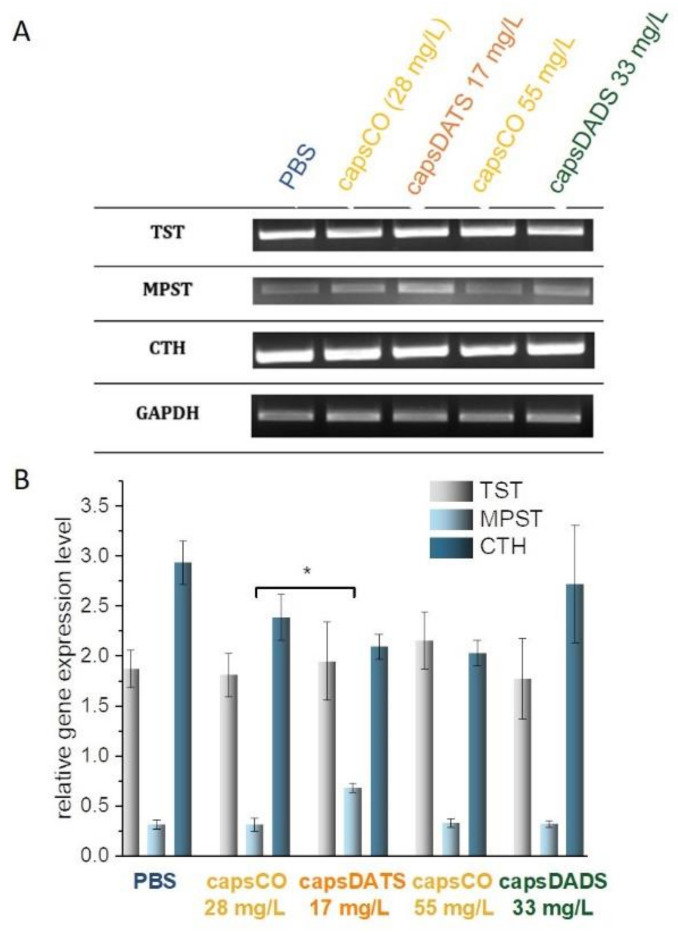
RT-PCR analysis of gene expression for TST, MPST and CTH. (**A**) Gene expression in the 4T1 cell line. The results are representative and obtained from 3 tests. (**B**) The relative gene expression level of TST, MPST and CTH in 4T1 cell line, presented as means +/− SD (n = 3). The densities of bands were normalized using the signal for the reference GAPDH gene. Significance estimated with the Mann–Whitney U-test in relation to the respective control groups (samples treated with PBS); * *p* < 0.05.

The DADS and DATS are produced from the decomposition of allicin present in garlic and have been studied extensively for their health benefits. In 2007, Benavides and co-workers [70] reported that DADS and DATS could be converted into H_2_S through a thiol, mainly glutathione, dependent mechanism. Liang et al. [10] verified this hypothesis and complemented the current knowledge with the mechanisms of DADS and DATS reactions and possible pathways of their transformations. Their results indicate that DADS can react rapidly with GSH via thiol-disulfide exchange to generate allyl mercaptan and S-allyl glutathione disulfide. The latter may undergo a similar thiol-disulfide exchange with another GSH, producing allyl mercaptan and GSSG. The results of our studies, summarized in Table 2, confirm the DADS metabolism pathway proposed by Liang et al. [10]. In the capsDADS-treated group, we observed an increase in glutathione levels, and under conditions of glutathione excess, DADS metabolism contributes to the increase in GSSG levels that we noted. The data derived by Liang and co-workers [10] indicated the existence of persulfide intermediates (GSSH and allyl polysulfides—ASSH) in the reaction between DADS and GSH. Highly reactive persulfides contain sulfane sulfur atoms in their structures. They are a new class of bioactive compounds as they have different reaction patterns leading to the release of this sulfur atom or its transfer to locally available acceptors such as cyanides, sulfites, glutathione, etc. We can see a reflection of this fact in the obtained results (Table 3), in which the activity (but not the expression, Figure 9) of TST in the capsDADS-treated group increases significantly as compared with the activity of this enzyme in the control group. The TST is responsible for transferring sulfur atoms from various donors (sulfane sulfur-containing compounds) to various acceptors [71]. In this case, the activities of the other two enzymes, MPST and CTH (Table 3) and their gene expression levels (Figure 9), as well as the level of sulfane sulfur, remain unchanged (Table 3). The MPST catalyzes the transfer of the sulfane sulfur atom from 3-mercaptopyruvate to various acceptors, producing sulfane sulfur-containing compound (e.g., thiosulfate) and also releases it as hydrogen sulfide [72]. The CTH is involved in sulfane sulfur generation in the cells [73].

According to Liang et al. [10], for DATS catabolism there are two possible thiol-disulfide exchange reaction pathways. The first one is the nucleophilic attack of GSH on allylic sulfur generating S-allyl glutathione disulfide and allyl persulfide, with the latter possibly releasing H_2_S, upon reduction by GSH. The second one is the nucleophilic attack of GSH on the central sulfur atom of DATS leading to the formation of allyl mercaptan and S-allyl glutathione trisulfide. Both of them, together with formed allyl persulfide and reactive thiols such as GSH, in the system may undergo further metathesis and redox reaction to form a series of glutathione- and allyl-polysulfides (G-(S)n-A and A-(S)n-A, n = 2–6). When an excessive amount of GSH is available, these compounds could eventually be reduced by GSH to produce H_2_S. All of the enzymes we studied (CTH, MPST and TST) participate in a series of reactions leading to the generation of hydrogen sulfide [40,74]. Thus, it was reasonable to assume that in the capsDATS-treated group, the levels of sulfurtransferase activity and/or expression level of their genes would be altered that was expected. According to data collected in Table 3, the activity of TST and CTH increased as compared with the control group, while the MPST activity decreased. Decreased MPST activity in cells (Table 3) implies an increase in the expression of the gene encoding this protein (Figure 9), which may reflect an increased cellular demand for this protein—likely related to an increase in the intensity of the processes in which it is involved. The expression levels of genes encoding the other two proteins remain unchanged (Figure 9). Changes in the activity of enzymes involved in the formation (CTH, MPST) and turnover (TST) of sulfane sulfur-containing compounds, as well as a decrease in sulfane sulfur level (Table 3), indicate that intensive polysulfide conversion processes occurs in cells treated with capsDATS. The results obtained remain in agreement with our expectations, so it can be concluded that both DADS and DATS are efficiently delivered to the cells, and the process of their encapsulation does not adversely affect the biological function they exhibit.

## 4. Conclusions

The studied HA-based nanocapsules are promising carriers addressing the important challenges related to (1) delivery of hydrophobic compounds (herein garlic oil pharmaceutically active components: DADS and DATS), (2) undesirable interactions of those compounds with body fluid components, and (3) instability of the number of chemicals under oxygen conditions or low pH. The results let us postulate that the HA-based nanocapsules may act as versatile delivery systems improving patients’ safety. We proved their long-term stability, which emphasizes the conclusion that regardless of the core material, HA-based systems are stable. Moreover, they show several advantageous features such as: lack of aggregations with serum components, reduced hemolysis effect, and stability in the presence of digestive system main enzymes and in low pH conditions, which indicate a great potential of HA-based formulation in various therapies. Additionally, we proved that HyC12 protects the encapsulated compounds against oxygen, which normally destabilizes the structures of numerous organic compounds, including studied DADS and DATS. Also, we showed that the encapsulation process itself does not alter the biological function of the tested compounds. We also proved that the ^1^H NMR technique can serve as a great tool for studying emulsions-like samples, especially with liquid cores.

Considering the anticancer properties of DADS and DATS, we showed that the anticancer activity of these substances is retained, and the encapsulation process can enhance the bioavailability of tested compounds by increasing their stability under environmental conditions. Thus, nanocapsules are a promising alternative to unfavorable organic solvents (e.g., DMSO) which are not indifferent to living cells.

## Figures and Tables

**Table 1:** Primer sequences used for reverse-transcription polymerase chain reaction (RT-PCR).

Genes *	Forward (F) and Reverse (R) Primers (5′ → 3′)	RT-PCR Product Size (bp)
CTH	F: CAGCAAGACCCGATGCAAAG R: CAAAGCAACACCTGCCACTC	304 [40,41]
MPST	F: AGCATTTATGAAGCCCGCCT R: CCTGGTCACTGTCGTCGTAG	420 [40,41]
TST	F: AACCTGGGCATAAGCAACGA R: GGTCCACCTTCTTGTCCTGG	460 [40,41]
GAPDH	F: GTCCCAGCTTAGGTTCATCAG R: TTTGGCTCCACCCTTCAAGT	404 [40,41]

* CTH—cystathionine γ-lyase (EC 4.4.1.1), MPST—sulfurtransferase 3-mercaptopyruvate (EC 2.8.1.2), TST—rhodanese (EC 2.8.1.1), GAPDH—gene encoding glyceraldehyde 3-phosphate dehydrogenase.

**Table 2:** The level of cysteine, oxidized (GSSG) and reduced (GSH) glutathione, total glutathione, and the ratio of reduced to oxidized form of glutathione in the 4T1 cells.

Group	Cysteine	GSH	GSSG	Total Glutathione	GSH/GSSG
	nmole/mg Protein				
Control	0.3 ± 0.2	23.6 ± 0.4	1.0 ± 0.2	25.6 ± 0.7	23.9 ± 3.3
capsCO (28 mg/L)	0.5 ± 0.1	25.0 ± 1.3	0.9 ± 0.2	26.8 ± 1.6	28.6 ± 3.8
capsDATS (17 mg/L)	2.2 ± 0.3 *	25.3 ± 1.5	0.6 ± 0.1	26.5 ± 1.6	41.2 ± 1.8 *
capsCO (55 mg/L)	0.3 ± 0.1	22.3 ± 1.5	0.9 ± 0.3	24.1 ± 2.1	25.5 ± 6.2
capsDADS (33 mg/L)	1.5 ± 0.5 *	60.0 ± 4.8 *	2.7 ± 0.6	65.4 ± 6.0 *	22.7 ± 3.7
capsDATS (33 mg/L)	1.2 ± 0.1 *	19.7 ± 2.9	0.12 ± 0.01 *	20.2 ± 4.0	174 ± 16 *

Data presented as mean +/− SD; n = 3–4. ‘Total glutathione in cells’ is denoted as the GSH + 2GSSG. The limit of detection for cysteine (CSH) and glutathione (GSH) in the RP-HPLC method is equal to 0.01 [nM/mL] and for the oxidized form of glutathione (GSSG) is 0.1 [nM/mL]. The limit of quantification for CSH and GSH is 0.1 [nM/mL], while for GSSG—1 [nM/mL] [49]. The appropriate capsules: capsCO vs. capsDADS/DATS have the same volumes of the oil phase, which results in different concentrations due to different densities of the used oils. Significance estimated with Mann–Whitney U test in relation to the respective control group; * *p* < 0.05.

**Table 3:** The level of sulfane sulfur and sulfurtransferases activity in the 4T1 cell line.

Group	Sulfane Sulfur	TST	MPST	CTH
	[nmol/mg Protein]	[nmol/mg Protein Min]		
control	185 ± 16	15.2 ± 1.9	76 ± 26	0.7 ± 0.1
capsCO (28 mg/L)	180 ± 6	14.6 ± 2.5	126 ± 33 ^#^	0.6 ± 0.1
capsDATS (17 mg/L)	158 ± 15 *	23.3 ± 3.6 *	99 ± 36 *	1.4 ± 0.5 *
capsCO (55 mg/L)	187 ± 12	12.2 ± 1.4 ^#^	133 ± 51 ^#^	1.1 ± 0.2 ^#^
capsDADS (33 mg/L)	176 ± 17	16.5 ± 3.3 *	133 ± 40	1.0 ± 0.3

The values represent the mean ± SD of 3 independent experiments, with each determination consisting of 4–15 assays. Significance estimated with the Mann–Whitney U-test in relation to the respective control group. ^#^ *p* < 0.05 for capsCO group (28 and 55 mg/L) versus control group, * *p* < 0.05 for capsDADS and capsDATS group versus control group. The appropriate capsule groups: capsCO vs. capsDADS/DATS have the same volume of the oil phase, which results in different concentrations due to different densities of the used oils.

## Data Availability

Data is contained within the article.

## Supplementary Materials

The following data are available online at https://www.mdpi.com/article/10.3390/nano11051354/s1, Figure S1: Hemolytic effects of tested emulsions: capsCO, capsDADS, capsDATS and free oils: the pictures of representative samples after the incubation with red blood cells (RBCs) for 5 min at 37 °C and the centrifugation at 5000 rpm for 5 min. Figure S2: Cell viability (analyzed by NRU and XTT assays) after the treatment by Hy-based capsules with CO cores. Table S1: Averaged diameters of studied samples: capsules incubated in PBS and serum.
