# Supplementary material for: Hyaluronic Acid-Based Nanocapsules as Efficient Delivery Systems of Garlic Oil Active Components with Anticancer Activity
Source: Nanomaterials (Basel). 2021 May 20;11(5):1354. doi: 10.3390/nano11051354 (PMC8160828; doi:10.3390/nano11051354)
Supplement: Supplementary file 1 [file nanomaterials-11-01354-s001.zip › nanomaterials-1225501-supplementary.pdf]

Electronic Supplementary Information

Hyaluronic Acid-Based Nanocapsules as Efficient Delivery Systems of Garlic Oil Active Components with Anticancer Activity

Małgorzata Janik-Hazuka [1], Kamil Kamiński [1], Marta Kaczor-Kamińska [2], Joanna Szafraniec-Szczęsny [3], Aleksandra Kmak [1], Hassan Kassassir [4,5], Cezary Watała [4], Maria Wróbel [2] and Szczepan Zapotoczny [1,*]

[1] Jagiellonian University, Faculty of Chemistry, Gronostajowa 2, 30-387 Krakow, Poland; mal.janik@uj.edu.pl (M.J.-H.); kaminski@chemia.uj.edu.pl (K.K.); ola.kmak@student.uj.edu.pl (A.K.)
[2] Jagiellonian University Medical College, Faculty of Medicine, Chair of Medical Biochemistry, Kopernika 7, 31-034 Krakow, Poland; marta.b.kaczor@uj.edu.pl (M.K.-K.); mtk.wrobel@uj.edu.pl (M.W.)
[3] Jagiellonian University Medical College, Faculty of Pharmacy, Department of Pharmaceutical Technology and Biopharmaceutics, Medyczna 9, 30-688 Krakow, Poland; joanna.szafraniec@uj.edu.pl (J.S.-S.)
[4] Medical University of Lodz, Department of Haemostasis and Haemostatic Disorders, Chair of Biomedical Sciences, Mazowiecka 6/8, 92-215 Lodz, Poland; hkassassir@cbm.pan.pl (H.K.); cezary.watala@umed.lodz.pl (C.W.)
[5] Institute of Medical Biology, Polish Academy of Science, Lodowa 106, 93-232 Lodz, Poland
* Correspondence: zapotocz@chemia.uj.edu.pl (S.Z.); Tel.: +48-12-686-2530

Results

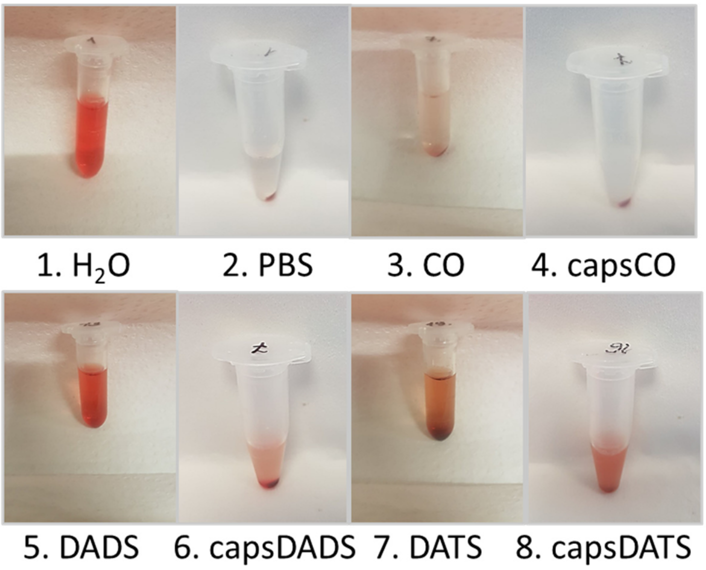

Figure S1. Hemolytic effects of tested emulsions: capsCO, capsDADS, capsDATS and free oils: the pictures of representative samples after the incubation with red blood cells (RBCs) for 5 min at 37 °C and the centrifugation at 5000 rpm for 5 min.

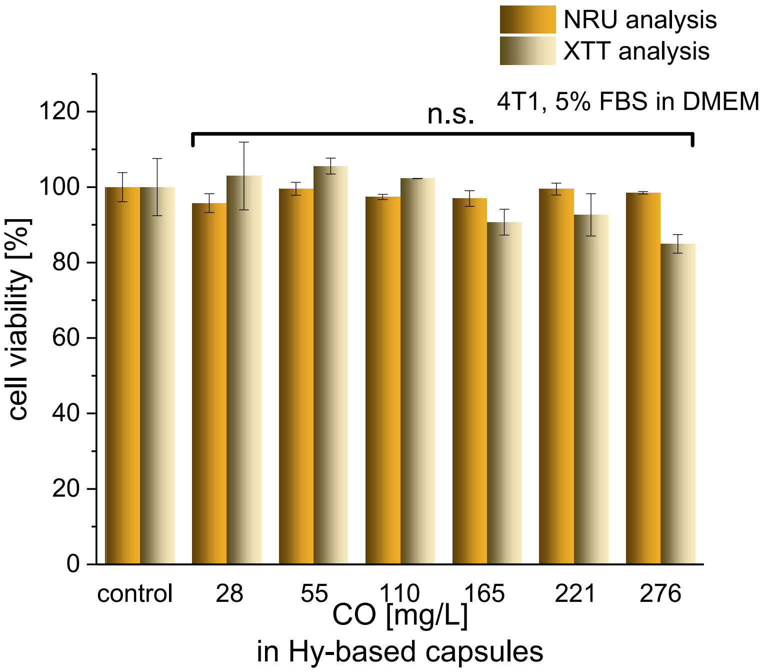

Figure S2. Cell viability (analyzed by NRU and XTT assays) after the treatment by Hy-based capsules with CO cores.

The data shown as mean +/- SD, (n=3) represent the amount of encapsulated and delivered compounds. The aqueous and oil phase were mixed in 1000:3 volume ratio. Significance estimated with the Mann-Whitney U-test; n.s., not significant.

Table S1. Averaged diameters of studied samples: capsules incubated in PBS and serum.

sample	size [nm]*	dispersity index (PdI)**
**capsCO-PBS**	520±25	0.243
**capsCO-serum**	48±11***	0.696***
**capsDADS-PBS**	468±19	0.358
**capsDADS-serum**	11±2***	0.582***
**capsDATS-PBS**	464±6	0.376
**capsDATS-serum**	12±2***	0.643***

Values represent the arithmetic mean ± standard deviation for 9 donors, with each determination consisting of 8 (incubated with PBS) and 24 (incubated with serum) assays. The incubation time was 1 h.

*Statistical significance, estimated with one-tailed Mann–Whitney U test or with unpaired Student's t-test (depending on the normality of data distribution), was: capsCO + PBS > capsCO + serum, $P_{1,\alpha} < 0.0001$; capsDADS + PBS > capsDADS+ serum, $P_{1,\alpha} < 0.0001$; capsDATS + PBS > capsDATS+ serum, $P_{1,\alpha} < 0.0001$;

** Statistical significance, estimated with one-tailed Mann–Whitney U test or with unpaired Student's t-test (depending on the normality of data distribution), was: capsCO + PBS < capsCO + serum, $P_{1,\alpha} < 0.0001$; capsDADS + PBS < capsDADS+ serum, $P_{1,\alpha} < 0.05$; capsDATS + PBS < capsDATS+ serum, $P_{1,\alpha} < 0.01$;

***change of average value due to multimodal distribution (see Figure 4 in the manuscript).
